# Potential Suicide Prophylactic Activity by the Fish Oil Metabolite, 4-Hydroxyhexenal

**DOI:** 10.3390/ijms23136953

**Published:** 2022-06-23

**Authors:** Hans O. Kalkman

**Affiliations:** Gänsbühlgartenweg 7, CH-4132 Muttenz, Switzerland; hans.kalkman@bluewin.ch; Tel.: +41-61-362-0110

**Keywords:** suicide prevention, fish oil, docosahexaenoic acid, NRF2, 4-hydroxyhexenal

## Abstract

Low levels of n-3 poly-unsaturated fatty acids (n-3 PUFAs) and high levels of n-6 PUFAs in the blood circulation are associated with an increased risk for suicide. Clinical studies indicate that docosahexaenoic acid (DHA, a n-3 PUFA found in fish-oil) displays protective effects against suicide. It has recently been proposed that the activation of the transcription factor NRF2 might be the pharmacological activity that is common to current anti-suicidal medications. Oxidation products from fish oil, including those from DHA, are electrophiles that reversibly bind to a protein ‘KEAP1’, which acts as the molecular inhibitor of NRF2 and so indirectly promotes NRF2-transcriptional activity. In the majority of publications, the NRF2-stimulant effect of DHA is ascribed to the metabolite 4-hydroxyhexenal (4HHE). It is suggested to investigate whether 4HHE will display a therapeutically useful anti-suicidal efficacy.

## 1. Introduction

Low levels of n-3 (omega-3) poly-unsaturated fatty acids (n-3 PUFAs) in plasma, serum and membranes of blood-cells are associated with an increased risk for suicide. For instance, in a prospective study of 100 suicide cases and 100 controls (patients injured by accidents), the levels of the n-3 PUFA eicosapentaenoicacid (EPA) in erythrocyte membranes were significantly lower in the suicide cases than in the control group [1]. In a retrospective case-controlled study consisting of 800 suicide deaths and 800 controls of US military, lower serum levels of the n-3 PUFA docosahexaenoic acid (DHA) related to higher risk of suicide death [2]. In a prospective study, suicide attempts were monitored over a period of two years in 33 medication-free depressed subjects. A low plasma ratio of DHA/total PUFA predicted suicide attempt [3]. In each of these studies the findings remained significant after adjustment for possible confounding factors. A postmortem study of the prefrontal cortex from adult depressed suicide victims (n = 20) and cardiovascular disease-free controls (n = 12) found significantly lower DHA levels among the suicide cases [4]. Finally, a prospective cross-sectional study in pregnant women (n = 234) provided indirect evidence for the importance of low n-3 PUFA levels in suicide risk. In this study [5], high levels of n-6 PUFA (arachidonic acid and adrenic acid) were associated with an increased risk of suicide. Since total PUFA is the sum of n-3 and n-6 PUFA, and because n-3 PUFA and n-6 PUFA compete for the same positions in phospholipids [6,7], a high n-6 level implies a low n-3 level.

Although DHA is clearly less effective than EPA as an antidepressant [8,9,10], the above-mentioned clinical studies indicate that DHA may have important protective effects against suicide. This raises the question about the possible mechanism of action. 

## 2. Prevention of Suicide by NRF2 Activation

Based on the molecular mechanisms of the anti-suicidal compounds lithium, clozapine and ketamine [11,12,13,14], it has recently been proposed that the activation of the transcription factor NRF2, might be the common underlying mechanism of action [15]. 

The activity of NRF2 is regulated via proteasomal degradation [16], which is promoted by phosphorylation via GSK3, and by dimerization with a molecule called “KEAP1” (Kelch-like erythroid cell-derived protein with CNC homology [ECH]-associated protein-1). The interaction between KEAP1 on NRF2 can be blocked by electrophilic compounds, such as sulforaphane (a product found in broccoli), and by medications such as dimethyl-fumarate or minocycline [16]. Such compounds increase the stability of NRF2, and thus promote the transcription of its target genes (for instance, NADPH:quinone oxidoreductase [NQO1], glutathione peroxidase-4 [GPX4], glutathione reductase-1 [GSR1], thioredoxin-1 [TXN1], sulfiredoxin-1 [SRXN1], heme oxygenase-1 [HO1] and sequestosome-1 [SQSTM1, also known as p65]) [17,18]. 

## 3. NRF2 Activation in Response to DHA

The anti-suicidal activity of n-3 PUFAs indicates that EPA and DHA might provoke NRF2-transcriptional activity. The chemical and enzymatic oxidation of EPA and DHA is known to yield a number of electrophiles. These include isoprostane (from EPA), neuroprostane (from DHA), 4-hydroxyhexenal (from n-3 PUFAs, such as α-linoleic acid, EPA and DHA) and (α, β)-unsaturated ketones, such as 17-oxo-DHA [19]. There is ample evidence from preclinical and clinical studies that these compounds indeed activate NRF2. 

For instance, in experiments in mouse skin and in cultured JB6 cells, 17-oxo-DHA provoked the nuclear localization of NRF2 and increased the expression of the target genes HO1 and NQO1, whereas such an effect was not shared by the non-electrophilic analog 17-hydroxy-DHA [20]. Notably, HO1 expression was absent in cells with a knockdown of NRF2 [20]. Chemical oxidation of EPA and DHA leads to isoprostanes and neuroprostanes, respectively [21]. In mouse hearts, isoprostanes and neuroprostanes interacted with KEAP1 and induced the nuclear accumulation of NRF2, which led to gene-transcription of HO1 [21]. A fish oil diet in mice (fish oil mainly contains EPA and DHA) increased HO1 expression in the aorta of wild-type mice, however, not in the aorta of NRF2^-/-^ mice [22]. In the wild-type strain, the levels of DHA and 4-hydroxyhexenal (4HHE) in the aorta were significantly increased [22]. In endothelial cells from human umbilical vein, incubation with DHA increased the intracellular levels of 4HHE, whereas 4HHE in a concentration-dependent manner increased the intra-nuclear levels of NRF2 and transcription of HO1 [23]. The major source for 4HHE in vascular tissue seems to DHA, not EPA [22]. In a similar experiment in a mouse adipocyte cell line, the incubation of EPA, DHA or 4HHE increased mRNA and protein levels of HO1, and this response again was absent after the knock-down of NRF2 [24]. The molecule 4HEE is generated by peroxidation of linolenic acid, EPA and DHA. It is an aldehyde with a relatively equal distribution between octanol and water distribution (Riahi et al. 2010 [PMID: 20858748]) and weak electrophilic reactivity towards the thiol group of cysteine (Cipollina, 2015 [PMID: 26339618]). The cardiovascular cytoprotective activity of n-3 fatty acids, brought about by transcription of NRF2 target genes, was subsequently confirmed by Lee et al. [25] and Wen et al. [26]. Moreover, the NRF2-mediated expression of target genes in response to DHA has been reported for human mammary epithelial cells [27], human epithelial cells [28], rat primary neurons [29] and rat hippocampus [30]. 

## 4. Discussion

Evidently, there is compelling evidence that oxidation products formed from fish oil, in particular from DHA, lead to the activation of the transcription factor NRF2. In a majority of cases the NRF2-transcriptional activity was ascribed to the production of 4-hydroxyhexenal (4HHE). This electrophilic compound forms adducts with certain conserved cysteine-residues of the NRF2-inhibiting molecule, KEAP1, leading to a disinhibition of NRF2 [16,17,31]. Interestingly, the suggestion that DHA is the major source of the 4HHE metabolite was formulated already 30 years ago [32]. It should be noted that a quite similar but more reactive compound, 4-hydroxy-nonenal (4HNE) is formed by the oxidation of n-6 PUFAs, such as linolenic acid, arachidonic acid and adrenic acid [31]. Therefore, one would expect an anti-suicide activity from treatment with n-6 PUFAs too. Apparently, this is not the case (see Introduction). When 4HHE and 4HNE react with cysteine residues of target proteins, the reaction with 4HHE is reversible, whereas the reversal reaction of the adduct between 4HNE and cysteine is much slower, and thus quasi-irreversible. The issue with electrophilic compounds is that they indiscriminately react with cysteine residues from numerous other peptides. For instance, 4HNE is known to bind to the cysteine residues of phosphatases, proteins that are functionally relevant for limiting the activity of MAP-kinase pathways [33,34]. Indeed, 4HNE has been shown to activate the JNK pathway, the p38 pathway and the MEK-ERK pathway, and so promotes pro-inflammatory responses [31,33,34]. In addition, 4HNE reacts with the cysteine residue of glutathione, and thus scavenges this important anti-oxidant molecule [34,35]. The functional outcome of this reaction is an activation of caspases, DNA fragmentation, mitochondrial damage and apoptosis [34,35,36,37]. Moreover, the loss of glutathione favors the continuous peroxidation of PUFAs [36] and triggers vicious processes that are thought to contribute to the development of chronic diseases [31,36,37]. It is remarkable that such deleterious effects are not described for 4HHE. Apparently, the balance between enhanced NRF2-driven transcription of anti-oxidant genes and the scavenging of glutathione is much better for 4HHE than for 4HNE. As mentioned in the introduction, suicide cases are characterized by low n-3 PUFA levels in blood and blood cells. A high intake of vegetable oil (rich in n-6 PUFAs and typical for Western diets) may be assumed to give rise to high levels of 4HNE, but low levels of 4HHE. Therefore, it would not be surprising that the prevention of suicide might already be achieved by avoiding diets that are rich in n-6 PUFAs.

A dysfunction of the AKT-GSK3β-NRF2 axis is thought to play a role in human neurological disorders, such as Parkinson’s disease, Alzheimer’s disease, amyotrophic lateral sclerosis and adrenoleukodystrophy [38,39] but also in mood disorders [40]. The pathophysiological processes involved in mood disorders, and in particular those involved in suicide, are still largely unknown. Suicide seems to be a two-stage process [41,42]. Suicide ideation is often associated with an inflammatory process for instance neurotropic pathogens, life-long stressors, allergies, autoimmunity or traumatic brain injury [15,43], whereas the actual attempt often involves increased irritability, hypervigilance or aggression [44]. Pro-inflammatory cytokines affect the metabolism of tryptophan [45], which alters neurotransmitter function in the brain and is thought to contribute to irritability and aggression [46]. These processes are conceivably ameliorated by the activation of the AKT-GSK3β-NRF2 pathway. NRF2-null mice display a particular profile of plasma lipid levels [47]. The lipid profile is quite similar to what has been noted in individuals who attempted suicide [48,49,50]. In this context, functional NRF2 protects unsaturated fatty acids against oxidative damage and is, therefore, relevant for the composition of mitochondrial membranes [39], endothelium function, including the function of the blood-brain barrier and cerebral perfusion [51] and myelin formation [39]. It is likely that these neurological processes play a role in the psychological aspects of suicide, and that the activation of NRF2 transcriptional activity may ameliorate these dysfunctional processes. 

As summarized in the introduction, a dietary supplementation with n-3 fatty acids seems to be beneficial. In a rat model of spinal cord injury, dietary supplementation with DHA or EPA normalized lipid peroxidation, normalized the low level of glutathione and increased the activity of the superoxide dismutase, glutathione peroxidase and catalase enzymes [52]. Moreover, in an in vitro assay of rat primary astrocytes exposed to oxidative stress (H_2_O_2_), both n-3 PUFAs concentration-dependently increased the level of glutathione and increased the levels of the NRF2-target genes [53]. These data, though limited, indicate that DHA and mixtures of DHA and EPA, stimulate NRF2-transcriptional activity. This may explain why fish oil displays a protective effect against suicide. Moreover, since 4HHE is arguably an important mediator of fish oil-induced NRF2 activation, it could be worthwhile to study if 4HHE itself provides useful anti-suicidal effects. 

## Data Availability

Not applicable.
